# Epilepsy

**Published:** 1892-06

**Authors:** Hattie C. Van Buren

**Affiliations:** Chatham, N. Y.


### EPILEPSY.

### Dr. McLaren's Paper in March Number.

Editor Homœopathic Physician:

In the issue of March there is given a report of Dr. McLaren's paper on Epilepsy, read before the I. H. A.

I would like to ask why he did not repeat Belladonna when it gave such a good result as relief for five weeks instead of introducing another remedy, viz., Calcarea? Did not Hahnemann teach that the effect of a drug was to be exhausted before prescribing another?

There was no opportunity given to see if Bell. would do more good.

Again, after giving the Calc. and producing "first a marked aggravation followed by an amelioration," when the efficacy of the one dose seemed completed, why were Hyoscyamus and Cina prescribed, when, as the author distinctly states, some of the indications calling for Calcarea remained? Would they not have disappeared if another dose of Calc. had been administered? The results finally demonstrated that Causticum was *the* remedy. Nevertheless, as the Doctor also states the approach of puberty complicated the case, would it not have all the more strongly called for a continuation of Bell. in the first place, or at least would it not have been strict Hahnemannism to have repeated the dose of Bell. before trying other remedies?

Hattie C. Van Buren, M. D.,

Chatham, N. Y.

